# Supplementary material for: MetaRibo-Seq measures translation in microbiomes
Source: Nat Commun. 2020 Jun 29;11:3268. doi: 10.1038/s41467-020-17081-z (PMC7324362; doi:10.1038/s41467-020-17081-z)
Supplement: Supplementary file 10 — Supplementary Data 7 [file 41467_2020_17081_MOESM10_ESM.zip › File2/Confidence_VeryHigh_Taxonomy/68483_out.krona.html]

Javascript must be enabled to view this page.

members
magnitude
magnitudeUnassigned
count
unassigned
taxon
rank

68483\_out

4

superkingdom
4
2

1239
phylum
4

186801
class
4

4
order
186802

family
1
31979

genus
1
1485


SRS047014\_contig\_number\_21771
1262777
species
1

family
3
186803

SRS143181\_contig\_number\_contig-100\_21882.21882
1

572511
1
genus


SRS063040\_contig\_number\_contig-100\_5243.107084
40520
1
species

genus
1
841

2049040

SRS142781\_contig\_number\_contig-100\_15699.58757
species
1
